# Bridging the Care Gap for People Living Alone With Dementia

**DOI:** 10.1093/geroni/igaf122.1725

**Published:** 2025-12-31

**Authors:** Heather Menne, Kate Singer, Emerson McSparran, Matt Nelson

**Affiliations:** Miami University, Oxford, Ohio, United States; Miami University, Oxford, Ohio, United States; Miami University, Oxford, Ohio, United States; Miami University, Oxford, Ohio, United States

## Abstract

Researchers, practitioners, and policymakers are drawing attention to the experiences of people living alone with dementia. Research in the US and UK describes the common characteristics and service needs of people who are living alone with dementia. Community-based providers are identifying and serving this often overlooked and complex population, and policymakers in federal and state governments are acknowledging the role of policies and programs to address any gaps in care for people living alone with dementia. Using data from the National Health and Aging Trends Study (NHATS), the Health and Retirement Study (HRS) and the National Survey of Older Americans Act Participants (NSOAAP), this analysis considers the role of home- and community-based services as one mechanism to support the ongoing independent, community-living of people living alone with dementia. Preliminary results indicate that less than 1% of the NHATS and HRS samples of adults aged 65 and older were living alone with dementia; in comparison, 1-6.4% of Older Americans Act (OAA) homemaker, case management, and home-delivered meal clients were living alone with dementia. Our findings demonstrate that community-based services play a role in supporting people living alone with dementia, and this is reinforced with the recent CMS Guiding an Improved Dementia Experience (GUIDE) Model which includes connections to community-based services. Encouragement of CMS GUIDE participating providers to connect older adults to qualified community-based providers and further investment in OAA and other community-based services are crucial steps to address the care and support needs of people living alone with dementia.

